# The 5’-nucleotidase S5nA is dispensable for evasion of phagocytosis and biofilm formation in *Streptococcus pyogenes*

**DOI:** 10.1371/journal.pone.0211074

**Published:** 2019-01-31

**Authors:** Marcel-Lino Dangel, Johann-Christoph Dettmann, Steffi Haßelbarth, Martin Krogull, Miriam Schakat, Bernd Kreikemeyer, Tomas Fiedler

**Affiliations:** Rostock University Medical Centre; Institute of Medical Microbiology, Virology, and Hygiene, Rostock, Germany; Oregon Health & Science University, UNITED STATES

## Abstract

5’-nucleotidases are widespread among all domains of life. The enzymes hydrolyze phosphate residues from nucleotides and nucleotide derivatives. In some pathobiontic bacteria, 5’-nucleotidases contribute to immune evasion by dephosphorylating adenosine mono-, di-, or tri-phosphates, thereby either decreasing the concentration of pro-inflammatory ATP or increasing the concentration of anti-inflammatory adenosine, both acting on purinergic receptors of phagocytic cells. The strict human pathogen *Streptococcus pyogenes* expresses a surface-associated 5’-nucleotidase (S5nA) under infection conditions that has previously been discussed as a potential virulence factor. Here we show that deletion of the S5nA gene does not significantly affect growth in human blood, evasion of phagocytosis by neutrophils, formation of biofilms and virulence in an infection model with larvae of the greater wax moth *Galleria mellonella* in *S*. *pyogenes* serotypes M6, M18 and M49. Hence, the surface-associated 5’-nucleotidase S5nA seems dispensable for evasion of phagocytosis and biofilm formation in *S*. *pyogenes*.

## Introduction

*Streptococcus pyogenes* (Group A *Streptococcus*) is a Gram-positive human pathogen primarily causing purulent infections of the skin (impetigo) and throat (pharyngitis, tonsillitis) and frequently also severe invasive or systemic diseases such as necrotizing fasciitis, sepsis or streptococcal toxic shock-like syndrome [1]. The list of newly identified factors contributing to virulence mechanisms of *S*. *pyogenes* is constantly growing. To date, more than 50 virulence factors have been described in *S*. *pyogenes* [2]. Recently, the 5’-nucleotidase S5nA has been added to that list of potential virulence factors [3].

5’-nucleotidases (5’NT) are enzymes hydrolyzing phosphate groups at the 5‘-end of ribose or deoxyribose in nucleotides or nucleotide-derivatives [4]. These enzymes are widely distributed among bacteria, plants and vertebrate tissues [4–6]. In bacteria cytoplasmic and periplasmic as well as membrane-associated 5’NT have been found [4]. Especially the membrane associated 5’NT are discussed to be associated with virulence in several pathogenic or pathobiontic bacteria such as *Vibrio cholerae* [7], *Pseudomonas aeruginosa* [8], *Staphylococcus aureus* [9] and *Streptococcus agalactiae* [10]. The immune-modulating effect of surface-exposed 5’NT of these bacteria is attributed to their ability to dephosphorylate adenosine phosphates. This can lead to a decreased concentration of the proinflammatory ATP and/or increased concentrations of adenosine, both mediating suppression of phagocytic activity via purinergic receptors on phagocytic cells [9–12]. Furthermore, in the non-pathogenic bacterium *Xylella fastidiosa*, an impact of a 5’NT on biofilm formation has been speculated [13].

In proteome analyses of *S*. *pyogenes* M49, a putative 5’NT with a leucine-proline-isoleucine-threnonine-glycine (LPITG) cell wall anchoring motif has been found in the surface-associated sub-proteome [14]. *In vitro* the 5’NT gene of *S*. *pyogenes* M1 SF370 is expressed in the early exponential growth phase but not in the stationary phase [15]. The presence of antibodies against the *S*. *pyogenes* 5’NT in serum samples of patients indicates that the enzyme is produced under infection conditions [16]. The 5’NT S5nA of *S*. *pyogenes* has been associated with immune evasion, as the addition of a recombinant S5nA of *S*. *pyogenes* M1 SF370 increased survival of the non-pathogenic *Lactococcus lactis* in human blood [3]. Whether S5nA actually contributes to virulence in *S*. *pyogenes* has not been investigated so far. In the work presented here we therefore analyzed virulence traits of S5nA mutants of three different *S*. *pyogenes* strains of the serotypes M6, M18 and M49.

## Materials and methods

### Bacterial strains and culture conditions

*S*. *pyogenes* serotype M6 strain K006 [17], M18 strain MGAS8232 [18], and M49 strain 591 were grown in Todd-Hewitt broth supplemented with 0.5% (w/v) yeast extract (THY medium) or Brain Heart Infusion (BHI) medium at 37 °C in 5% CO_2_ enriched ambient air in standing cultures. *S*. *pyogenes* deletion mutants were cultivated in THY medium containing 30 mg/L kanamycin. *S*. *pyogenes* strains carrying pIB184 derivatives were grown in THY containing 5 mg/L erythromycin. *Escherichia coli* DH5α was grown in Lysogeny Broth (LB) at 37 °C under ambient air. *E*. *coli* strains harbouring pSF151 [19] or pUC18Erm1 [20] derivatives were grown in LB containing 50 mg/L kanamycin or 300 mg/L erythromycin, respectively.

### Construction of recombinant vectors and *S*. *pyogenes* strains

For the construction of *S*. *pyogenes* 5’-nucleotidase gene knockout mutants, the upstream and downstream flanking regions of S5nA genes of *S*. *pyogenes* M6 K007 (M6_Spy0695 in the reference genome of *S*. *pyogenes* M6 MGAS10394), *S*. *pyogenes* M18 MGAS8232 (SpyM18_0933) and *S*. *pyogenes* M49 591 (Spy49_0686 in the reference genome of *S*. *pyogenes* M49 NZ131) were PCR-amplified and ligated into the pUC18Erm1 vector [20]. The kanamycin resistance gene *aphA* was amplified from vector pSF151 and ligated between the flanking regions. The resulting plasmid was electroporated into *S*. *pyogenes* M6 K006, M18 MGAS8232 and M49 591, respectively. Transformants were selected on THY agar plates with the respective antibiotics. Single crossover mutants were kanamycin- and erythromycin-resistant while double crossover deletion mutants were kanamycin-resistant but erythromycin-sensitive. No polar effects have to be considered as the S5nA gene is not part of an operon. For generation of complementation strains the M18 S5nA gene including promoter region was PCR-amplified and ligated into the shuttle vector pIB184 [21] via ApaI and BamHI restriction sites. Primers used in this study are listed in Table 1.

**Table 1:** Oligonucleotides.

Primer name	Oligonucleotide sequence (5`→ 3`)	Use
FR1_fw	ATTTGTCGACAAAATCTCCTAATAAGTT	Amplification of upstream and downstream flanking regions of S5nA genes
FR1_re	TTTAAAGGATCCATGCCAGACTTAAT	
FR2_fw	TAAATCTGCAGCTTATCTTCATA	
FR2_re	GGAAAGTCGACCGTTTACTAGTAATCGA	
aphA_fw	GCGTCGACGCTACCAAGACGAAGAGGATG	Amplification of kanamycin resistance gene *aphA*
aphA_re	GCGTCGACCTAAAACAATTCATCCAGTAA	
comp_fw	GAGCGGGCCCAATAATGTTCTAG	Amplification of S5nA genes for complementation
comp_re	CCAGGATCCATTAGATCATCTAG	
20Sp5’NT_001qF	CGGCGCTCTTGATAATACCG	S5nA qPCR
20Sp5’NT_002qR	CAGAGTTAGCAGGACTGGC	
5SqF	AGCGACTACCTTATCTCACAG	5S rRNA qPCR
5SqR	GAGATACACCTGTACCCATG	

Underlined letters indicate restriction sites.

### Quantitative real-time PCR

For RNA isolation, bacteria were grown in 20 ml of THY, harvested in the exponential growth phase (optical density at 600 nm = 0.3) and quickly frozen in liquid nitrogen. As described by Pappesch et al. [22], RNA was isolated with the Direct-zol RNA MiniPrep Kit (Zymo Research), subsequent acid phenol:chloroform:isoamyl alcohol (125:24:1) extraction and TURBO DNase treatment. cDNA was generated with the SuperScript first-strand synthesis system for RT-PCR (Thermo Fisher Scientific). SYBR green (Thermo Fisher Scientific)-based quantitative real time PCR was carried out on a ViiA Real-Time PCR System (Applied Biosystems). The 5S rRNA gene was used as a housekeeping gene. Primers used for S5nA and 5S cDNA detection are listed in Table 1.

### Measurement of 5’-nucleotidase activity

5’-nucleotidase activity was determined from bacteria growing exponentially in THY medium at an optical density at 600 nm of 0.5 (exponential growth between OD600 of 0.2 to 0.7 for all strains, see S1 Fig). For that purpose, bacteria from 20 ml culture were harvested by centrifugation. Pellets were washed in physiological NaCl solution, suspended in 450 μl of a buffer containing 50 mM Tris (pH 6.5), 2 mM MgCl_2_ and 1 mM adenosine-5’-monophosphate (AMP) and incubated for 30 min at 37 °C. As controls, on the one hand bacteria were incubated in buffer without AMP and on the other hand the reaction mixture was incubated without bacteria. Subsequently, bacteria were pelleted by centrifugation and free phosphate in the supernatant was determined. For that purpose, 400 μl supernatant were mixed with 400 μl ammonium-vanadate solution (21 mM in 0.28 M HNO_3_) and 400 μl ammonium-molybdate solution (40 mM in 1.25 M H_2_SO_4_), incubated for 10 min at room temperature and extinction was measured at 405 nm. Phosphate concentrations were calculated based on a calibration curve. The calibration curve was generated with dipotassium phosphate solutions covering concentrations from 12.5 to 1000 μM. Values of AMP-free controls were subtracted from the other values. Controls without bacteria did not contain detectable amounts of inorganic phosphate.

### Biofilm cultivation and quantification

For biofilm assays, bacterial overnight cultures in THY were suspended in fresh BHI supplemented with 0.5% glucose, adjusted to 10^4^ CFU/ml and inoculated into 96-well microtiter plates. Wells were coated overnight at 4 °C with 2 μg/well human collagen I, collagen IV, or fibronectin (Sigma) in PBS. After incubation for 24, 48 or 72 h as standing cultures at 37 °C in a 5% CO_2_ / 20% O_2_ atmosphere, the biofilms were quantified in a Spectramax M2 plate reader after staining with crystal violet as described previously [17].

### Blood survival/Growth assay

The blood survival assays were performed as described previously [23]. Briefly, overnight cultures of *S*. *pyogenes* were inoculated into fresh THY medium and grown to the exponential growth phase. Exponential growth was observed between OD600 of 0.2 to 0.7 for all strains (S1 Fig). Bacteria were harvested by centrifugation at an OD600 of 0.3 to 0.4, set to an optical density at 600 nm of 0.25, and further diluted 1:10,000 in PBS. The viable counts of this suspension were determined. Twenty microliters of the suspension were incubated with either 480 μl of heparinized human blood at 37 °C with rotation. After 3 h, viable counts in the blood samples were determined and correlated with the inoculum to calculate multiplication factors.

### Quantitative phagocytosis assay

The quantitative phagocytosis assay was performed with primary neutrophils as described previously [22]. Briefly, neutrophils were isolated from human citrated blood using PolymorphPrep (Progen Biotechnik GmbH, Heidelberg) following the manufacturer’s instructions. Neutrophils were suspended in RPMI medium. 10^7^ neutrophils and opsonized bacteria were mixed in a 1:1 ratio. After 30 min of co-incubation, the viable counts of the bacteria were determined and correlated with a mock control of bacteria mixed with an equal volume of RPMI medium instead of neutrophils.

### Ethics approval statement

The protocol for the collection of human blood for the blood survival assays and the isolation of neutrophils was approved by the Ethikkommission an der Medizinischen Fakultät der Universität Rostock (Ethics Committee vote: A 2014–0131). The experiments were conducted in accordance with the ICH-GCP guidelines. Oral informed consent was obtained from all subjects.

### *Galleria mellonella* infection model

Larvae of the greater wax moth *Galleria mellonella* were obtained from Reptilienkosmos (Niederkrüchten, Germany). Infection experiments were carried out as described elsewhere [24]. In short, *S*. *pyogenes* strains were grown overnight in THY, washed twice in a 0.9% NaCl solution and suspended in 0.9% NaCl to a final concentration of 1.5-2x10^8^ CFU/ml. Larvae with a weight of 150–200 mg were inoculated with 10 μl of this bacterial suspension, resulting in an infection dose of 1.5–2 x 10^6^ CFU/larva. Bacteria were injected into the hemocoel of the larvae between the last pair of legs using a microapplicator (World Precisions Instruments, Sarasota, USA) and a fine dosage syringe (Omnican-F, 0.01 ml–1 ml, 0.30x12 mm, B. Braun AG, Melsungen, Germany). As a control, larvae were mock inoculated with 10 μl of a 0.9% NaCl solution. Survival of the larvae was observed for seven days. Larvae were regarded dead when they did not move upon repeated physical stimulation with tweezers.

## Results

5’NT have been described to contribute to virulence in several bacteria [3, 7–10]. S5nA, a recombinant 5’NT of *S*. *pyogenes* has been shown to increase survival of *L*. *lactis* in the presence of phagocytes in vitro [3]. Therefore, we aimed to elucidate whether deletion of the S5nA gene actually affects the virulence of *S*. *pyogenes*. For this purpose the S5nA genes were replaced by a kanamycin resistance gene in three different *S*. *pyogenes* strains, i.e. the serotype M49 strain 591 (reference sequence ORF spy49_0686c), the M18 serotype strain MGAS8232 (reference sequence ORF spyM18_0933) and the M6 serotype strain K006 (reference sequence ORF M6_Spy0695). Furthermore, complementation strains expressing the S5nA gene from a pAT19-based plasmid under the control of the native promoter were constructed.

To assess whether the S5nA gene deletions led to a general growth deficiency of the bacteria, growth of all strains in complex laboratory medium THY was measured. Neither the mutants nor the complementation strains had any significant growth deficiencies as compared to the cognate wild type strains in THY medium. The respective specific growth rates in the exponential phase of growth and optical densities after overnight growth are listed in Table 2. The full growth curves are shown in S1 Fig.

**Table 2:** Growth of *S*. *pyogenes* wild type, S5nA deletion and complementation strains in THY medium^a^.

Strain	Specific growth rate μ in h^-1^			Optical density at 600 nm after 24 h		
	WT	ΔS5nA	ΔS5nA::S5nA	WT	ΔS5nA	ΔS5nA::S5nA
**K006 (M6)**	0.95 ± 0.10	0.96 ± 0.07	1.01 ± 0.08	1.7 ± 0.17	1.7 ± 0.17	1.7 ± 0.25
**MGAS8232 (M18)**	0.69 ± 0.09	0.66 ± 0.06	0.91 ± 0.09*	1.7 ± 0.19	1.6 ± 0.17	2.0 ± 0.18*
**591 (M49)**	0.80 ± 0.13	0.84 ± 0.08	0.84 ± 0.06	2.1 ± 0.35	2.1 ± 0.10	2.2 ± 0.16

^a^Data represent mean values ± standard deviations of five independent experiments;

*marginally significant difference compared to WT (p<0.05, Student’s t-test, n = 5)

The absence or presence of S5nA gene transcripts in exponentially growing wild type, mutant, and complementation strain cells was detected via qPCR with 5S rRNA as a housekeeping reference gene. As expected, no S5nA gene transcripts were detectable in the mutant strains whereas S5nA transcripts were present in all wild type and complementation strains (see S1 Table). In the complementation strains, however, the relative abundance of S5nA gene mRNA was notably higher than in the wild-type strains, likely because in these strains the gene is transcribed ectopically from a plasmid that is present in more than one copy per cell.

**Fig 1:**
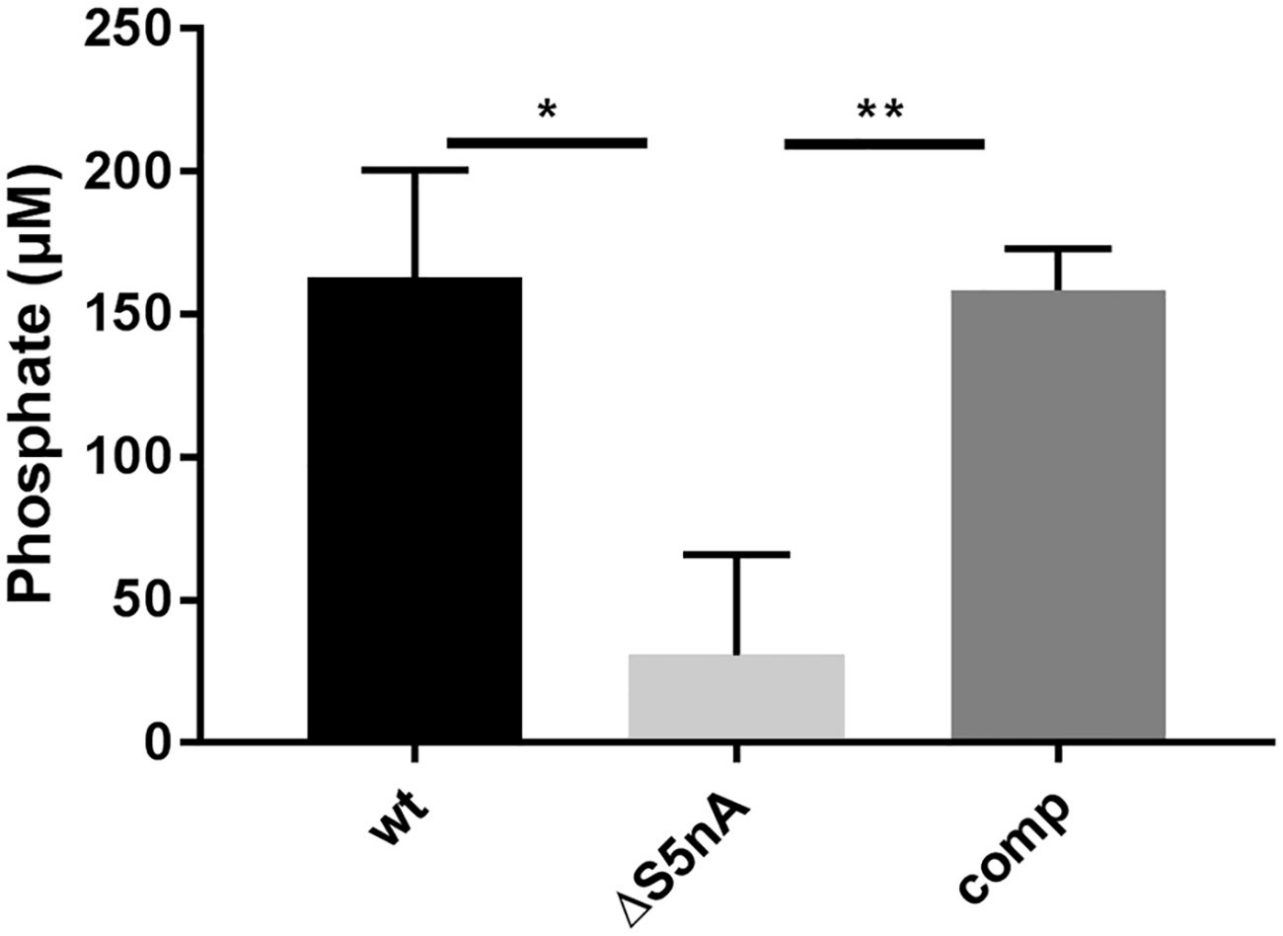
Surface 5’NT activity. Bacteria of wild type (wt), S5nA deletion (ΔS5nA) and complementation (comp) strains were harvested in the exponential growth phase and incubated with AMP as substrate and phosphate release was measured. Phosphate concentrations are shown as means with standard deviation (n = 3 biological replicates, *p<0.05, **p<0.01, unpaired two-tailed t-test).

Since it has been proposed that S5nA of *S*. *pyogenes* contributes to virulence by degrading AMP (and to some extent ADP) and consequently increases levels of the anti-inflammatory adenosine [3], we analyzed whether the deletion of the above-mentioned S5nA gene of *S*. *pyogenes* M49 actually leads to a loss of AMP dephosphorylation activity on the surface of the bacteria. For that purpose, exponentially growing bacteria of wild type and mutant strains were harvested at an optical density of 0.5, washed, and incubated with adenosine-monophosphate (AMP) in a phosphate-free buffer. After 30 min, phosphate concentrations were measured. About 30 μM of inorganic phosphate was hydrolyzed from AMP by the S5nA deletion mutant while wild type and complementation strains released about 150 μM of inorganic phosphate from 1 mM of AMP in the same time (Fig 1).

**Fig 2:**
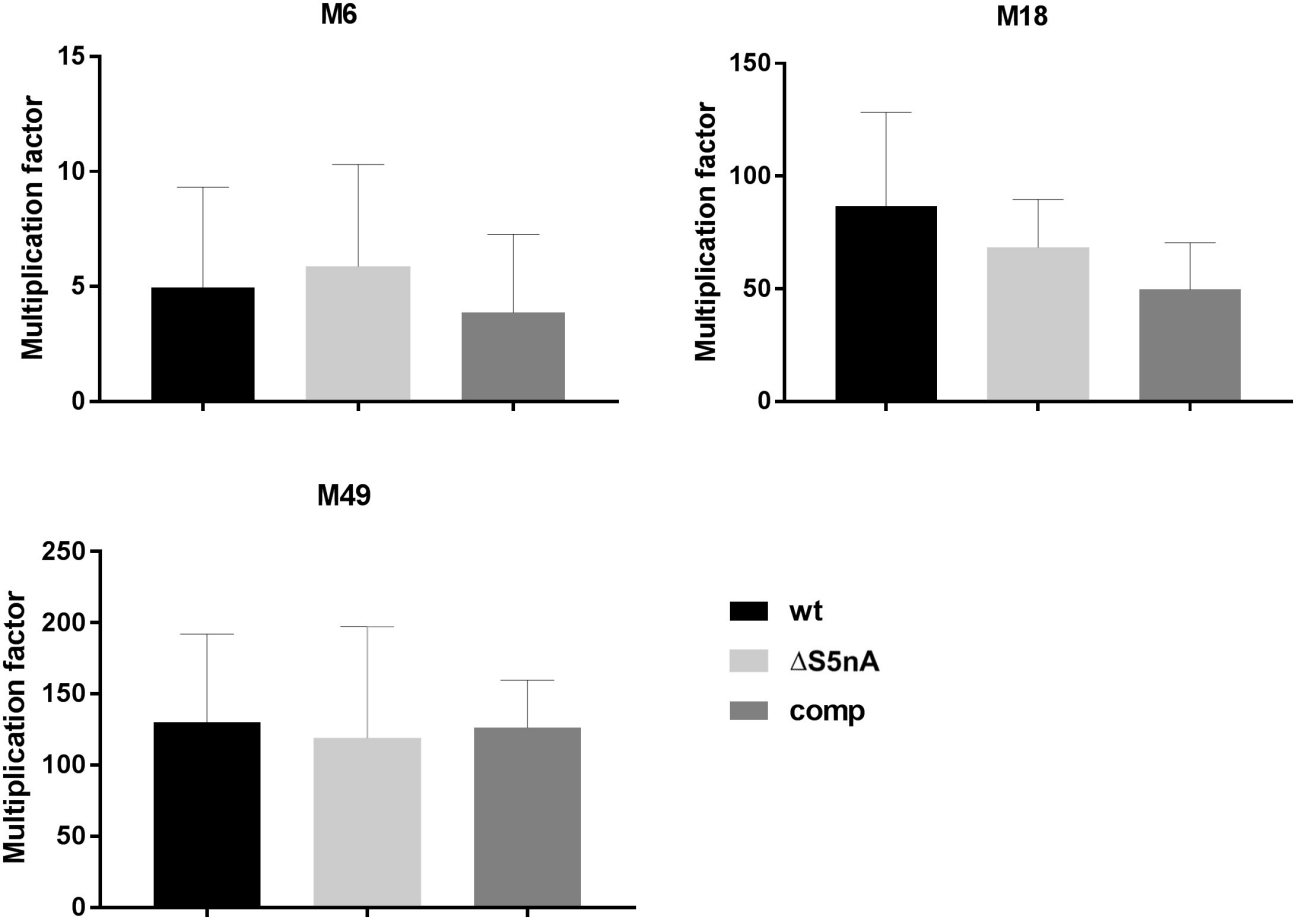
Growth in human blood. Bacteria were incubated in heparinized human blood for three hours. Viable counts of wild type (wt), S5nA deletion (ΔS5nA), and complementation (comp) strains before and after the incubation were used to calculate multiplication rates. Multiplication rates are shown as means with standard deviation (n = 5 biological replicates, no significant differences, unpaired two-tailed t-test).

*S*. *pyogenes* S5nA activity releases adenosine from AMP and ADP [3]. Adenosine triggers purinergic receptors of phagocytic cells leading to a suppression of their phagocytic activity [9, 11, 12]. Therefore, we tested the growth of the S5nA gene deletion strains in blood by inoculating the exponentially growing bacteria in fresh heparinized human blood and comparing viable counts of the bacteria before and after 3 h of incubation. As can be seen in Fig 2, the S5nA gene deletion strains had no serious disadvantage in the blood environment. Although in the M18 and M49 background the S5nA gene deletion strains had a slightly lower multiplication rate than the wild types, these differences were not statistically significant (Fig 2). Furthermore, at least in the M18 background, the complementation strain was not able to reverse this tendency, indicating that there is no S5nA specific effect. These data indicate that S5nA does not significantly contribute to innate immune evasion of *S*. *pyogenes* in blood.

**Fig 3:**
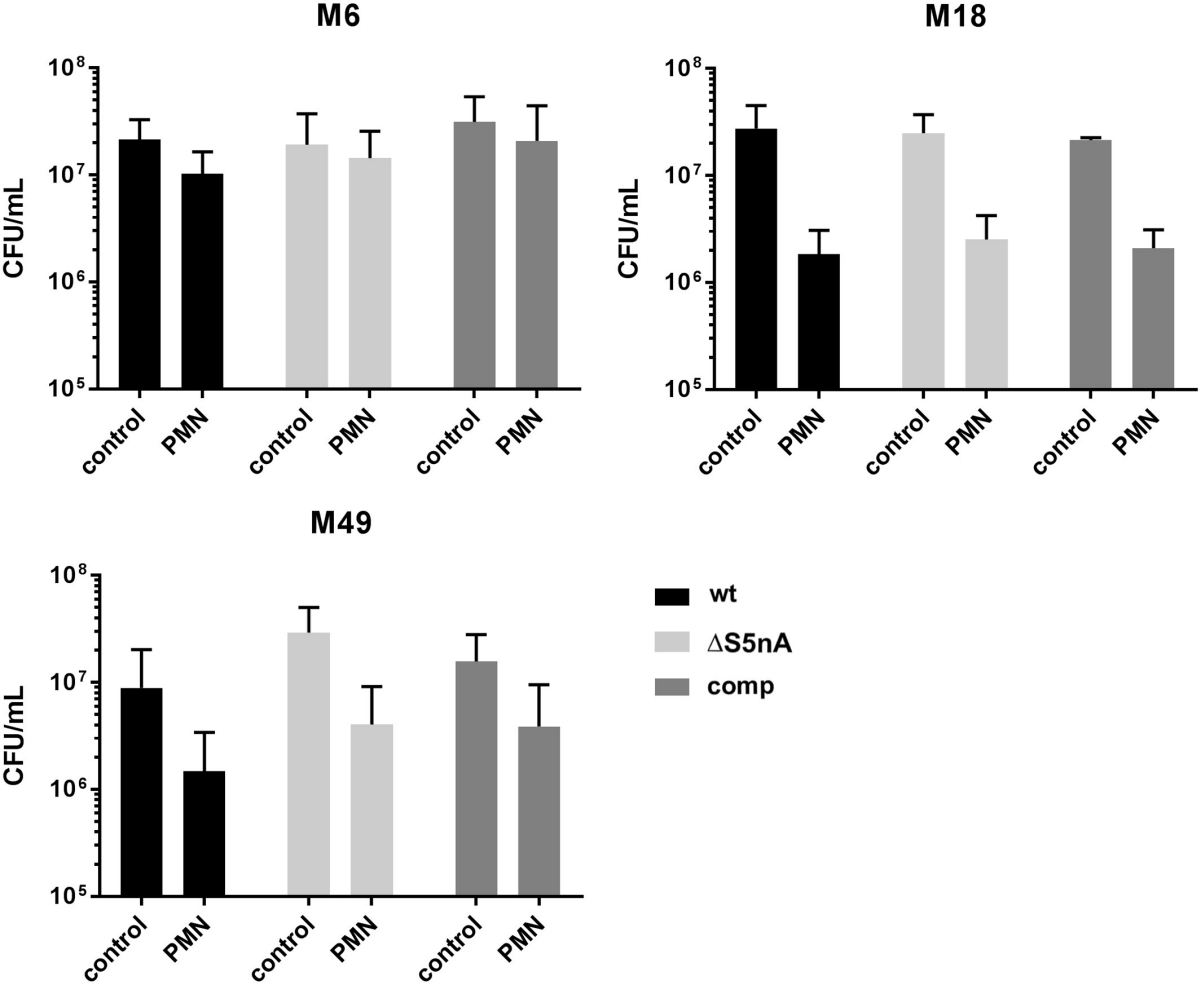
Phagocytosis by neutrophils. Phagocytic clearance was measured by determining viable counts of wild type (wt), S5nA deletion (ΔS5nA) and complementation (comp) strains after 30 min of incubation of opsonized bacteria in RPMI medium in the absence (control) or in the presence of neutrophils (PMN) at an MOI of 1. Viable counts are shown as means with standard deviation (n = 4 biological replicates).

To further validate these results, we investigated whether S5nA gene deletion renders *S*. *pyogenes* more prone to phagocytosis. As neutrophils account for the majority of phagocytic cells in blood, opsonized bacteria were mixed 1:1 with freshly isolated neutrophils and viable counts were determined after 30 min of incubation. While neutrophils were able to reduce the viable counts of M18 and M49 strains by about one order of magnitude within 30 min, the M6 strains were rather resistant to phagocytic clearance by the neutrophils. However, in accordance with the observations in the blood survival experiments neither of the S5nA deletion strains was killed more efficiently than its cognate wild type and complementation strain (Fig 3).

**Fig 4:**
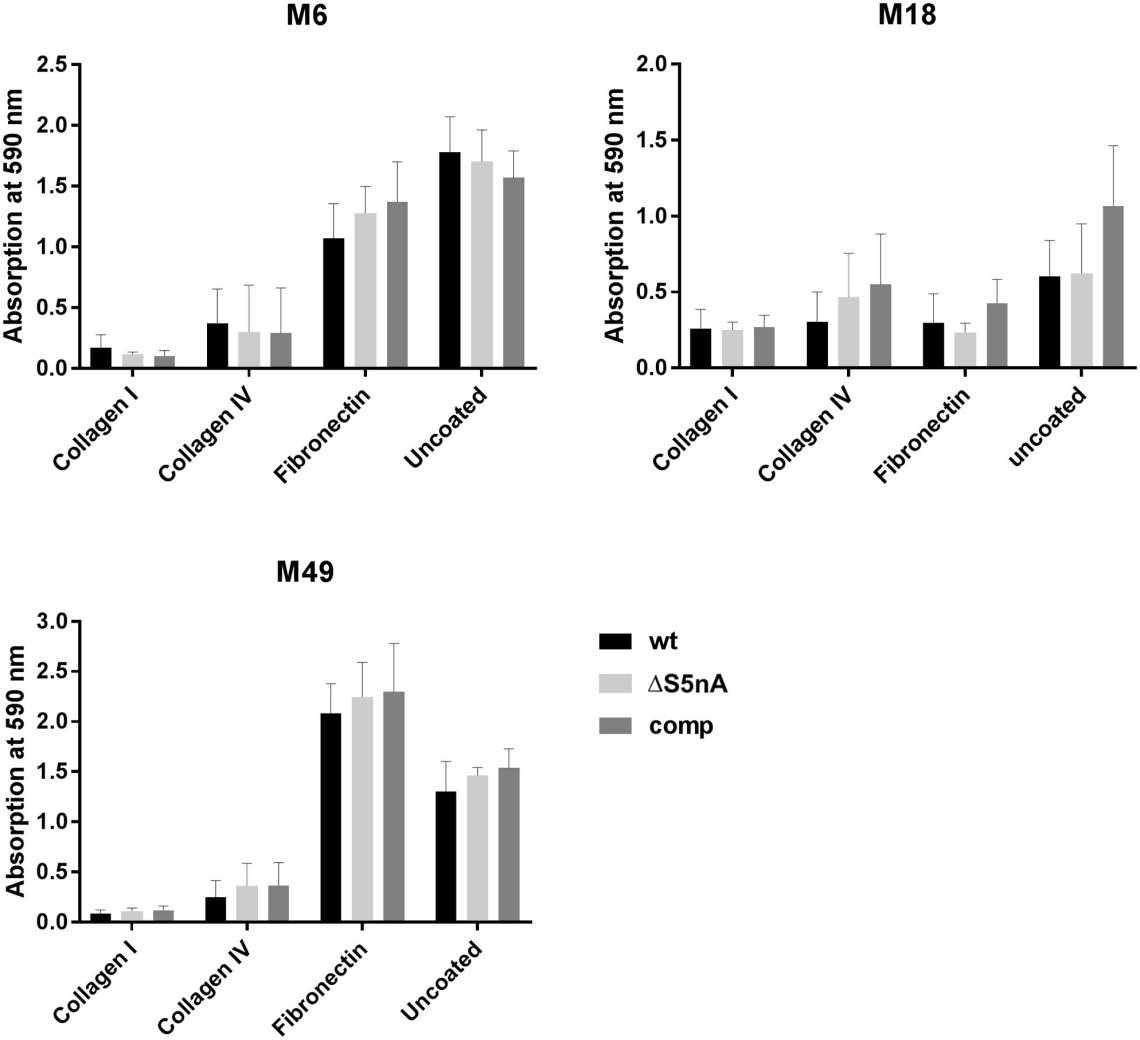
Biofilm formation. Bacteria were grown in 96-well plates for 72 h in BHI medium containing 0.5% glucose. Biofilm masses of wild type (wt), S5nA deletion (ΔS5nA) and complementation (comp) strains were determined via crystal violet staining. No significant differences in absorbance at 592 nm were detected between wt, 5SnA deletion and complementation strains when grown on the same surface. Data are shown as means with standard deviation (n = 5 biological replicates, unpaired two-tailed t-test).

Besides the implication of 5’NT in the inhibition of phagocytosis in several bacteria [3, 9, 10, 25, 26], for *X*. *fastidiosa* an involvement of 5’NT in biofilm formation has been proposed [13]. Therefore, we also investigated the impact of the loss of S5nA on biofilm formation of *S*. *pyogenes*. Since *S*. *pyogenes* strains differ in their ability to form biofilms on abiotic or protein-coated surfaces [27, 28], we analyzed biofilm formation on polystyrene and also on collagen I, collagen IV and fibronectin coated surfaces. Biofilm masses were assessed after 24 h, 48 h and 72 h of growth in 96-well plates. While the general biofilm-formation capacity was clearly strain-dependent, no significant differences in biofilm masses were observed between wild type, S5nA gene deletion mutant and complementation strain of the same serotype (for 72 h incubation values see Fig 4; for 24 and 48 h values see S2 Fig).

**Fig 5:**
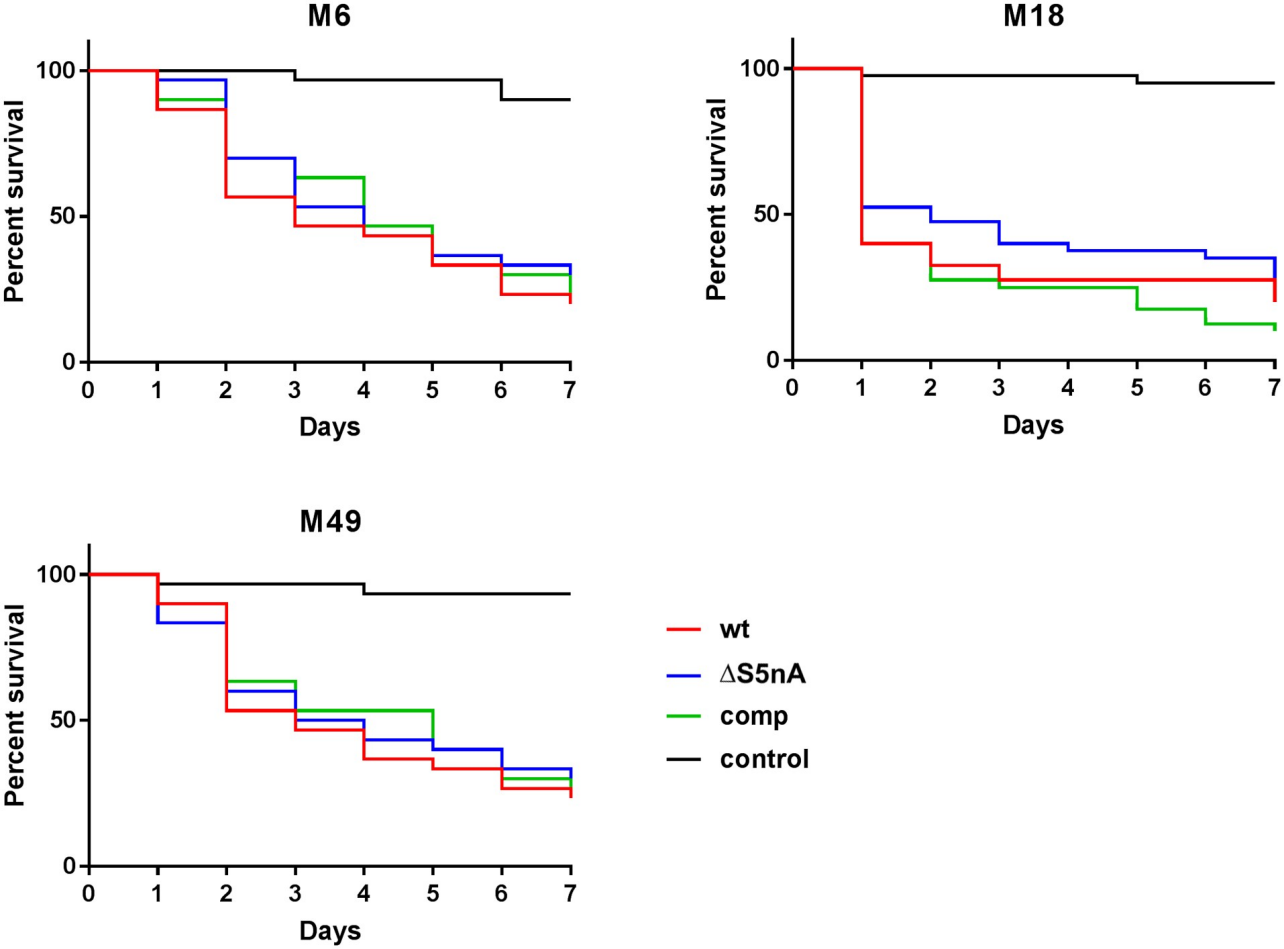
*Galleria mellonella* infection model. Survival of *Galleria mellonella* larvae after infection with wild type (wt), S5nA deletion (ΔS5nA) and complementation (comp) strains and a mock control inoculated with PBS instead of bacteria (control). No significant differences occurred in survival of the larvae infected with wildtype, S5nA deletion or complementation strains within one serotype (n = 30 biological replicates, log-rank test).

Finally, the virulence of the S5nA gene deletion strains was tested in an infection model using the larvae of the greater wax moth *Galleria mellonella*. For this purpose the animals were infected with defined doses of bacteria and survival of the larvae was observed for 10 d post infection. Again only serotype-dependent differences were observed. The M18 strain was most virulent in the larvae with about 50% of the animals dead 24 h after infection, while 50% of the larvae infected with the M6 or M49 serotype strains survived for two to three days (Fig 5). However, there were no significant differences in the survival rates of the larvae after infection with S5nA deletion strains in comparison with wild type and complementation strains of the same serotype (Fig 5). Altogether, these data indicated that S5nA is rather dispensable for virulence of *S*. *pyogenes*.

## Discussion

5’NT have been associated with virulence of certain bacteria, as it has been shown that they hydrolyze phosphate residues of adenosine phosphates and hence can interfere with the immunologically relevant ATP/adenosine ratio in human infections. This may happen via production of the anti-inflammatory adenosine and/or degradation of the pro-inflammatory ATP, both acting on phagocytic cells via purinergic receptors [9, 11, 12, 29–33].

*S*. *pyogenes* encodes the surface-associated 5’NT S5nA that has its highest expression levels in the exponential growth phase and that is immunogenic, as antibodies against *S*. *pyogenes* S5nA can be found in the serum of patients after *S*. *pyogenes* infections [14–16]. Although it has been shown that S5nA of the *S*. *pyogenes* M1 strain SF370 allows *L*. *lactis* to survive in human blood when added as a recombinant enzyme [3], the implication of the enzyme in virulence of *S*. *pyogenes* has not yet been investigated. Since the *S*. *pyogenes* S5nA dephosphorylates ADP and AMP but not ATP, the effect on *L*. *lactis* survival is mediated by increasing the adenosine concentration rather than lowering ATP levels [3]. This mode of action has also been described for the 5’NT of other streptococci such as *S*. *agalactiae* and *S*. *suis* [10, 25]. The 5’NT of *Streptococcus sanguinis*, in contrast, directly degrades ATP into adenosine [34]. In *S*. *agalactiae* and *S*. *suis* the deletion of the 5’NT genes rendered the bacteria more prone to phagocytosis, consequently leading to a decreased survival in blood and decreased virulence in mouse (*S*. *agalactiae*) and piglet (*S*. *suis*) infection models [10, 25]. In *Streptococcus equi* subsp. *zooepidemicus* two nucleases with 5’NT activity have been shown to contribute to survival of the bacteria in neutrophil extracellular traps (NET) by degrading the NET DNA. The respective deletion mutants were also less virulent in a mouse infection model [26]. However, none of these effects were observed in the *S*. *pyogenes* S5nA gene deletion strains investigated here. The S5nA deletion strains had no survival deficiencies in human blood nor were they more efficiently killed by neutrophils. This is also reflected in the *G*. *mellonella* infection model, where the larvae did not survive in a higher proportion when infected with the deletion strains in comparison to those infected with wild type strains. There was however a residual phosphate release from AMP in the S5nA deletion mutant of *S*. *pyogenes* M49 (Fig 1). According to sequence data in the NCBI database, none of the proteins with an LPXTG cell wall anchoring motif encoded in the *S*. *pyogenes* M49 genome harbors a 5’-nucleotidase domain structure. The residual AMP dephosphorylating activity might therefore result from cytosolic nucleotide phosphatases leaking from defective cells or, less likely, a so far unrecognized cell wall anchored protein with minor 5’-nucleotidase activity.

It is not clear however if 5SnA does not contribute to evasion of phagocytosis in *S*. *pyogenes* wild type bacteria at all or if the bacteria are able to compensate for the loss of S5nA. *S*. *pyogenes* is equipped with numerous other virulence factors mediating enhanced resistance to phagocytosis, including complement inhibitors, leucocidal toxins, immunoglobulin binding and degrading enzymes, NET degrading nucleases, and others (for an overview see review by Walker et al., 2014 [35]) that might be able to compensate for the loss of S5nA. The residual AMP-dephosphorylation activity measured in the 5SnA deletion strains—if attributable to a so far unrecognized minor 5’-nucleotidase—might also contribute to a possible compensation. Furthermore, although phagocytosis resistance and survival/growth in human blood seem not largely dependent on S5nA in *S*. *pyogenes* it cannot be excluded that S5nA mediated increase in adenosine levels in *S*. *pyogenes* infections has an impact on other immunological mechanism, as adenosine also acts as a signaling molecule regulating cytokine release of regulatory T cells and other immune cells [36, 37]. A mammalian infection model might uncover such effects. The *G*. *mellonella* model—although well established for investigations on virulence of *S*. *pyogenes* and other bacterial pathogens [24, 38–41]–naturally comes with certain limitations as the insect immune systems only resembles the innate immune mechanisms of humans. Purinergic receptors of the adenosine-binding P1-type receptors have been described to be present on insect cells [42]. To our knowledge however the role of adenosine in regulation of phagocytosis by hemocytes of *G*. *mellonella* has not been investigated yet.

Apart from the impact of 5’NT on the phagocytosis resistance of several bacteria, there is also indirect evidence for an association of 5’NT with biofilm formation in some bacteria. In *X*. *fastidiosa* it has been shown that a cytosolic 5’NT is highly expressed during the initial phase of biofilm formation. The authors speculate an impact of the 5’NT on the phosphorylation status of quorum-sensing molecules that might affect biofilm formation [13]. Biofilm formation capability is remarkably heterogeneous among *S*. *pyogenes* strains [43]. An association between biofilm formation capacity and certain pilus types has been described [17, 44]. Pilus proteins are encoded in the “fibronectin-binding, collagen-binding, T-antigen (FCT)” region in the *S*. *pyogenes* genome. To date nine different FCT types have been described that differ in composition and number of genes included [45, 46]. The M18 and M49 serotype strains investigated here carry an FCT type 3 region. Biofilm formation in FCT-3 strains is described to be inhomogeneous and dependent on environmental conditions. The M6 serotype strain K007, in contrast, contains an FCT type 1 region that is described to be associated with strong biofilm formation that is less depending on environmental conditions [17, 44]. In this respect our data are in accordance with the literature, as the FCT type 1 M6 strain formed strong biofilms on uncoated and fibronectin-coated plastic surfaces. As it has been described, for the FCT type 3 strains investigated here the biofilm formation capacity was variable. While the M49 strain was able to form stable biofilms on fibronectin and uncoated plastic, the M18 was barely able to form biofilms on any of the surfaces. In any case, in neither of the strains did the deletion of the S5nA gene have a significant impact on biofilm formation.

## Conclusions

Our study shows that deletion of the 5’NT encoding gene S5nA does not affect *S*. *pyogenes* with respect to phagocytosis by neutrophils, growth of the bacteria in human blood, survival rates of the *G*. *mellonella* larvae in the infection model or biofilm formation capacity in three different strains covering the serotypes M6, M18 and M49. Hence, although an immunomodulatory capacity of the *S*. *pyogenes* S5nA was demonstrated by Zheng and colleagues [3], for the evasion of phagocytosis and biofilm formation this enzyme seems to be dispensable for *S*. *pyogenes*.

## Supporting information

S1 Fig Growth curves of *S*. *pyogenes* M6, M18, and M49 (lower row) wild type (wt), S5nA gene deletion (ΔS5nA), and complementation (comp) strains in THY medium. The data are shown as means of n = 5 biological replicates.
(TIF)

S2 Fig Biofilm masses of *S*. *pyogenes* M6 (upper row), M18 (middle row), and M49 (lower row) wild type (wt), S5nA gene deletion (ΔS5nA), and complementation (comp) strains after 24 h (left column) and 48 h (right column) as determined via photometry after crystal violet staining. Shown are average values and standard deviations of n≥3 independent experiments.
(TIF)

S1 Table qPCR results (Cycle threshold [Ct] means, n = 3) of S5nA mRNA quantification with 5S rRNA as control. (DOCX)
