# Supplementary figures and images for: The 5’-nucleotidase S5nA is dispensable for evasion of phagocytosis and biofilm formation in Streptococcus pyogenes
Source: PLoS One. 2019 Jan 31;14(1):e0211074. doi: 10.1371/journal.pone.0211074 (PMC6354987; doi:10.1371/journal.pone.0211074)

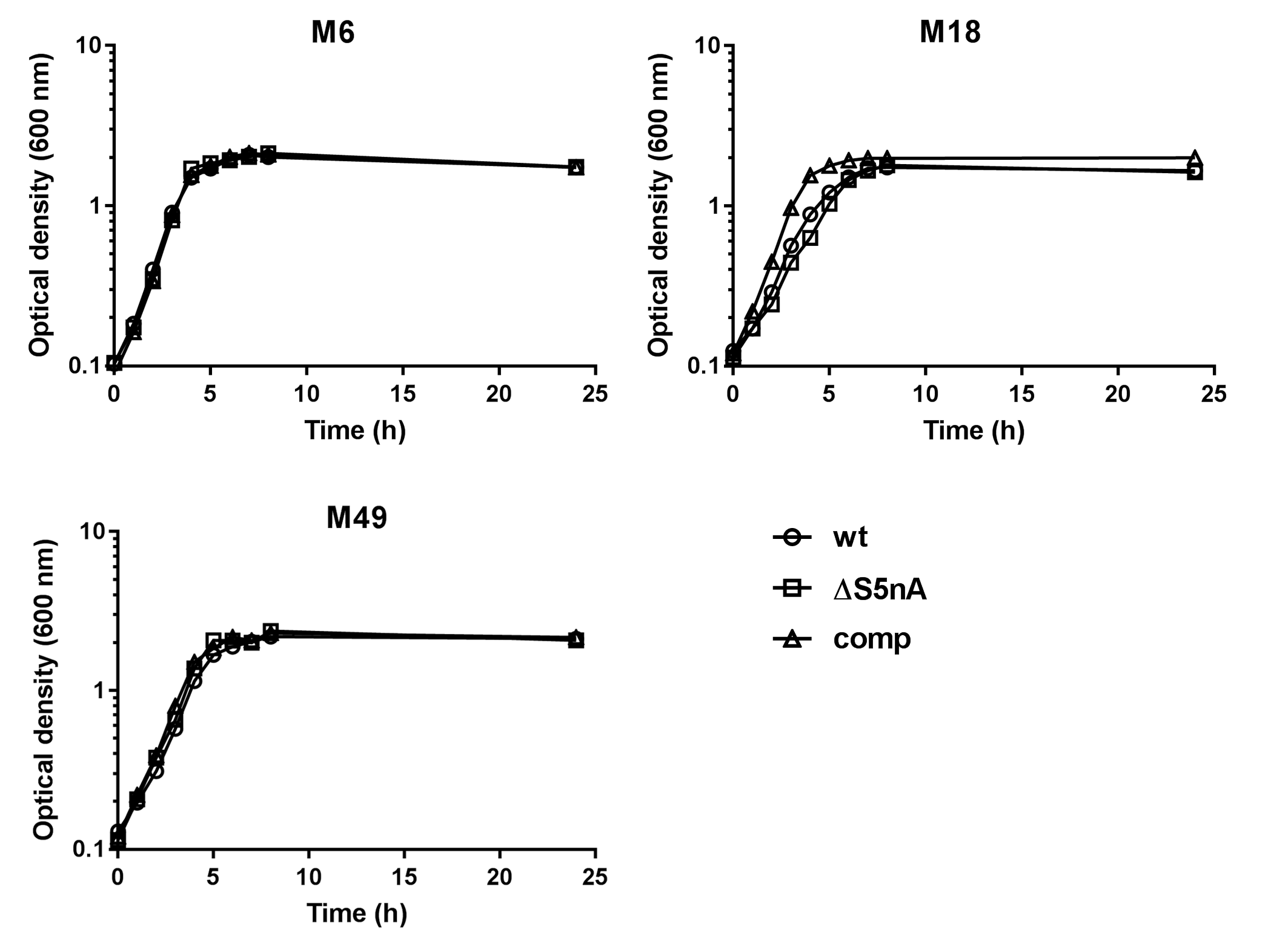

Supplement: S1 Fig — The data are shown as means of n = 5 biological replicates. (TIF) [file pone.0211074.s001.tif]

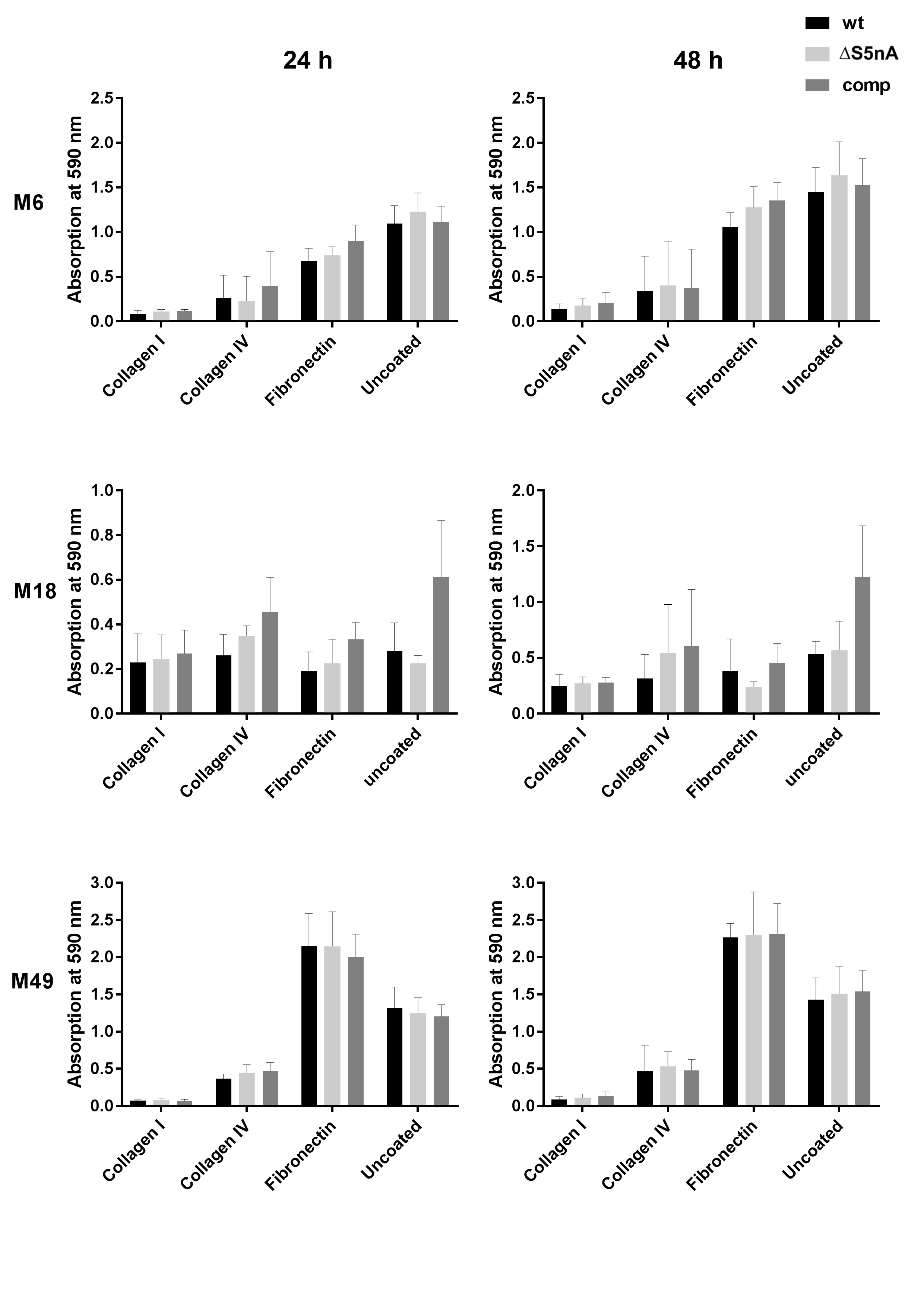

Supplement: S2 Fig — Shown are average values and standard deviations of n≥3 independent experiments. (TIF) [file pone.0211074.s002.tif]
