# Supplementary material for: The 5’-nucleotidase S5nA is dispensable for evasion of phagocytosis and biofilm formation in Streptococcus pyogenes
Source: PLoS One. 2019 Jan 31;14(1):e0211074. doi: 10.1371/journal.pone.0211074 (PMC6354987; doi:10.1371/journal.pone.0211074)
Supplement: S1 Table — (DOCX) [file pone.0211074.s003.docx]

| Srain | Mean  Ct (5S rRNA) | Standard Deviation  Ct (5S rRNA) | Mean  Ct (S5nA) | Standard Deviation  Ct (S5nA) | ΔCt | ΔΔCt | 2^-ΔΔCt^ |
| --- | --- | --- | --- | --- | --- | --- | --- |
| M6 wt | 18.55 | 0.07 | 25.15 | 0.31 | 6.60 | 0.00 | 1 |
| M6 ΔS5nA | 18.37 | 3.93 | 37.12 | 2.06 | 18.75 | 12.5 | 0.00 |
| M6 comp | 18.50 | 0.13 | 21.90 | 0.28 | 3.40 | -3.20 | 9.18 |
| M18 wt | 18.63 | 0.39 | 32.20 | 0.74 | 13.56 | 0.00 | 1 |
| M18 ΔS5nA | 17.87 | 0.05 | nd | - | - | - | - |
| M18 comp | 19.00 | 0.20 | 26.77 | 4.60 | 7.77 | -5.79 | 55.48 |
| M49 wt | 18.19 | 3.55 | 23.31 | 0.14 | 5.12 | 0.00 | 1 |
| M49 ΔS5nA | 18.44 | 0.24 | 36.30 | 3.13 | 17.87 | 12.75 | 0.00 |
| M49 comp | 18.35 | 0.60 | 17.15 | 0.38 | -1.19 | -6.31 | 79.55 |
| Neg. Kontrolle | 35.59 | - | - | - | - | - | - |
